# *Mycobacterium abscessus* Complex Infections in Humans

**DOI:** 10.3201/2109.141634

**Published:** 2015-09

**Authors:** Meng-Rui Lee, Wang-Huei Sheng, Chien-Ching Hung, Chong-Jen Yu, Li-Na Lee, Po-Ren Hsueh

**Affiliations:** National Taiwan University Hospital Hsin-Chu Branch, Hsin-Chu, Taiwan (M.-R. Lee, W.-H. Sheng, C.-C. Hung, C.-J. Yu, L.-N. Lee, P.-R. Hsueh);; National Taiwan University Hospital, Taipei, Taiwan (M.-R. Lee, W.-H. Sheng, C.-C. Hung, C.-J. Yu, L.-N. Lee, P.-R. Hsueh)

**Keywords:** Mycobacterium abscessus complex, Mycobacterium abscessus, Mycobacterium massiliense, Mycobacterium bolletii, multidrug resistant, nontuberculous, mycobacteria, outbreaks, cosmetic procedures, nosocomial, transmission, taxonomy, nomenclature, clinical disease, bacteria, identification methods

## Abstract

*Mycobacterium abscessus* complex comprises a group of rapidly growing, multidrug-resistant, nontuberculous mycobacteria that are responsible for a wide spectrum of skin and soft tissue diseases, central nervous system infections, bacteremia, and ocular and other infections. *M. abscessus* complex is differentiated into 3 subspecies: *M. abscessus* subsp. *abscessus, M. abscessus* subsp. *massiliense*, and *M. abscessus* subsp. *bolletii.* The 2 major subspecies, *M. abscessus* subsp. *abscessus* and *M.* *abscessus* subsp. *massiliense*, have different *erm*(41) gene patterns. This gene provides intrinsic resistance to macrolides, so the different patterns lead to different treatment outcomes. *M. abscessus* complex outbreaks associated with cosmetic procedures and nosocomial transmissions are not uncommon. Clarithromycin, amikacin, and cefoxitin are the current antimicrobial drugs of choice for treatment. However, new treatment regimens are urgently needed, as are rapid and inexpensive identification methods and measures to contain nosocomial transmission and outbreaks.

## Abstract 2

New treatments, rapid and inexpensive identification methods, and measures to contain nosocomial transmission and outbreaks are urgently needed.

Mycobacteria are divided into 2 major groups for the purpose of diagnosis and treatment: *Mycobacterium tuberculosis* complex, which comprises *M. tuberculosis,* and nontuberculous mycobacteria (NTM), which comprise all of the other mycobacteria species that do not cause tuberculosis. NTM can cause pulmonary disease resembling tuberculosis, skin and soft tissue infections (SSTIs), central nervous system infections, bacteremia, and ocular and other infections (*1*,*2*). Over the past decade, the number of NTM disease cases worldwide has markedly increased (*3*,*4*), and the upsurge cannot be explained solely by increased awareness among physicians and advances in laboratory methods (*3*).

*M. abscessus* complex is a group of rapidly growing, multidrug-resistant NTM species that are ubiquitous in soil and water (*1*). Species comprising *M. avium* complex (MAC) are the most common NTM species responsible for disease; however, infections caused by *M. abscessus* complex are more difficult to treat because of antimicrobial drug resistance (*5*). *M. abscessus* complex is also resistant to disinfectants and, therefore, can cause postsurgical and postprocedural infections (*2*,*5*). Although *M. abscessus* complex most commonly causes SSTIs and pulmonary infections, the complex can also cause disease in almost all human organs (*2*,*5*). To improve our understanding of *M. abscessus* complex infections, we reviewed the epidemiology and clinical features of and treatment and prevention measure for diseases caused by the organisms as well as the taxonomy and antimicrobial susceptibilities of these organisms.

## Search Strategy and Selection Criteria

We performed a PubMed search for *M. abscessus* complex articles published during January 1990–December 2014, using the following search terms: *M. abscessus*, *M. abscessus* subsp. *abscessus*, *M. abscessus* subsp. *bolletii*, *M. abscessus* subsp. *massiliense*, *M. massiliense*, *M. bolletii*, and nontuberculous mycobacteria. Only articles published with abstracts in English were selected.

## Taxonomy and Epidemiology

### Bacterial Classification

**Figure 1:**
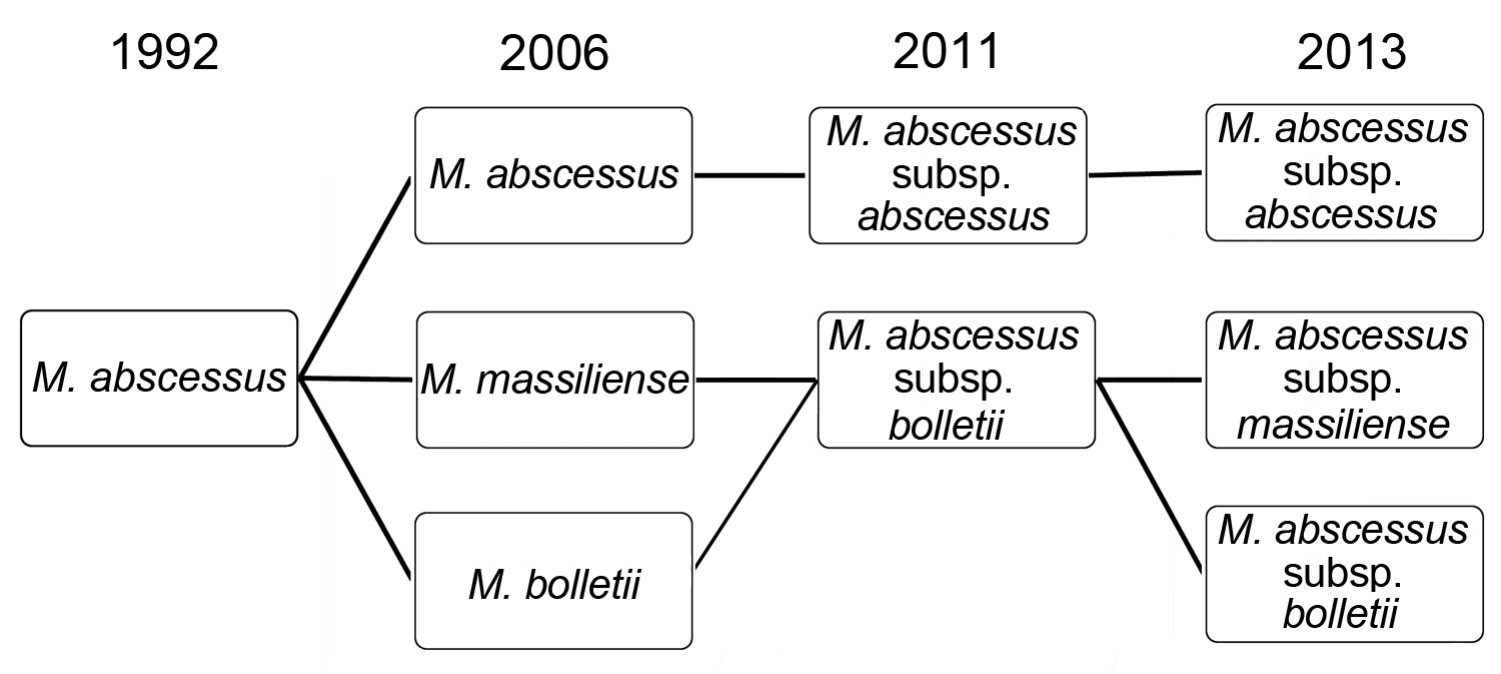
Serial changes in the nomenclature and taxonomic classification of *Mycobacterium abscessus* complex, 1992–2013.

*M. abscessus* was first isolated from a knee abscess in 1952 (*1*). *M. abscessus* and *M. chelonae* were originally considered to belong to the same species (“*M. chelonei*” or “*M. chelonae*”), but in 1992, *M. abscessus* was reclassified as an individual species (*1*). After *M. abscessus* was recognized as an independent species, new subspecies, including *M. massiliense* and *M. bolletii*, were discovered. Debate has ensued over whether *M. massiliense* and *M. bolletii* should be reunited to form one subspecies, *M. abscessus* subsp. *bolletii* (*6*). It is hoped that the debate will be settled as a result of findings from several recent studies that clearly demonstrated, by genome comparison, that *M. abscessus* complex comprises 3 entities: *M. abscessus* subsp. *abscessus*, *M. abscessus* subsp. *massiliense*, and *M. abscessus* subsp. *bolletii* (*7*–*11*). Serial changes in the taxonomic classification and nomenclature of *M. abscessus* complex, from 1992 to 2013, are shown in Figure 1.

*M. abscessus* subsp. *bolletii* is recognized as a rare pathogen with a functional inducible erythromycin ribosome methyltransferase (*erm*) (41) gene. In most *M. abscessus* subsp. *abscessus* mycobacterium, this gene leads to macrolide resistance. *M. abscessus* subsp. *massiliense* has been proposed to have a nonfunctional *erm*(41) gene, leading to macrolide susceptibility and a favorable treatment outcome for infections (*7*–*11*).

### Laboratory Identification

**Figure 2:**
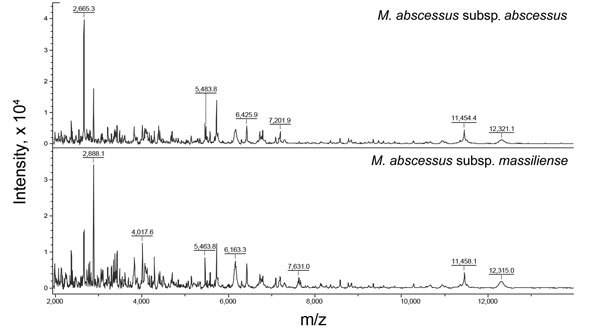
Spectrum of *Mycobacterium abscessus* subsp. *abscessus* and *M. abscessus* subsp. *massiliense* created by matrix-assisted laser desorption/ionization time-of-flight mass spectrometry Biotyper system (Microflex LT; Bruker Daltonik GmbH, Bremen, Germany). The absolute intensities of the ions are shown on the *y-*axis, and the masses (m/z) of the ions are shown on the *x-*axis. The m/z values represent the mass-to-charge ratio.

Definitive diagnosis of *M. abscessus* complex infection in humans is invariably determined by the isolation of *M. abscessus* complex from clinical specimens. The correct subspecies identification of *M. abscessus* complex has traditionally relied on phenotypic methods (e.g., biochemical testing for the utilization of citrate) to distinguish them from closely related species like *M. chelonae* (*1*). However, this method is not accurate enough to differentiate between the 2 main subspecies of the complex. Instead, *rpoB* gene–based sequencing is a more reliable method for correctly identifying *M. abscessus* complex to the subspecies level (*10*). However, because of the limited differences between the subspecies of *M. abscessus* complex, some researchers have questioned the accuracy of identification results from the sequencing of a single gene, especially the *rpoB* gene (*10*). Many schemes have been used in an attempt to accurately differentiate between subspecies, such as multilocus gene sequence typing, sequencing of the *erm* gene, and matrix-assisted laser desorption/ionization time-of-flight mass spectrometry (Figure 2) (*10*,*12*). Nonetheless, because the taxonomic classification is still changing, the debate over the optimal identification method will probably also continue.

## Disease Burden

The global isolation and epidemiology of *M. abscessus* complex are diverse. Furthermore, due to limitations in correct and detailed species identification, previous epidemiologic studies often referred to *M. abscessus* complex as *M. chelonae/abscessus* group or rapidly growing mycobacteria (*13*). In the United States, *M. abscessus/chelonae* complex infections are secondary only to MAC infections, compromising 2.6%–13.0% of all mycobacterial pulmonary infections across various study sites. This percentage correlates to an annual prevalence of <1 *M. abscessus*/*chelonae* pulmonary infections per 100,000 population, but the prevalence is increasing (*13*). *M. abscessus* complex is especially prevalent in East Asia. For example, in Taiwan, *M. abscessus* complex comprises 17.2% of all clinical NTM isolates, which correlates to 1.7 cases/100,000 population (*4*). According to current studies, the proportion of *M. abscessus* subsp. *massiliense* and *M. abscessus* subsp. *abscessus* is about the same among all clinical isolates (*12*). *M. abscessus* subsp. *bolletii* is rarely isolated (*7*).

## Clinical Diseases

### Respiratory Tract Infections

**Figure 3:**
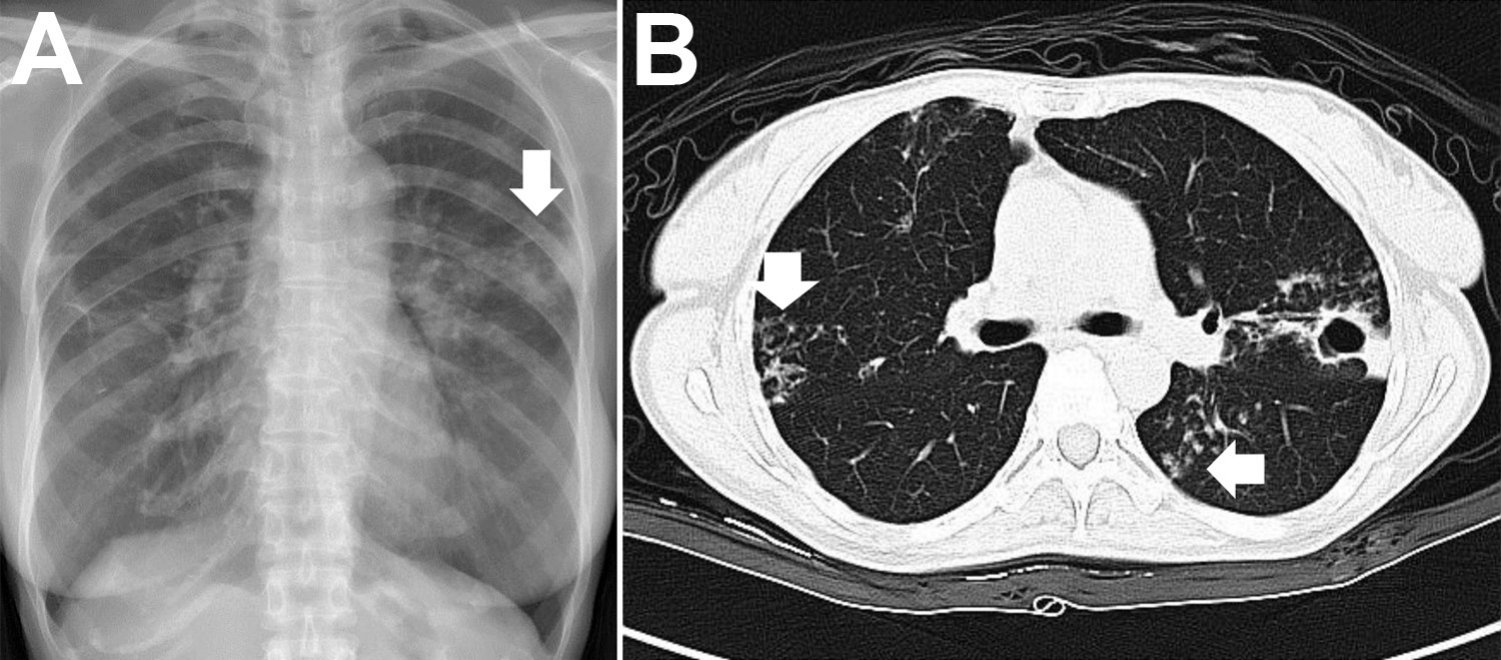
Chest radiograph (A) and computed tomography scan (B) images for a patient with pulmonary disease due to *Mycobacterium abscessus* subsp. a*bscessus*. A) The arrow indicates a cavity with surrounding consolidation over the left upper lung. B) Vertical arrow indicates bronchiectasis; horizontal arrow indicates nodules.

*M. abscessus* complex can cause pulmonary disease, especially in vulnerable hosts with underlying structural lung disease, such as cystic fibrosis, bronchiectasis, and prior tuberculosis (*2*). *M. abscessus* complex pulmonary disease usually follows an indolent, but progressive, course, causing persistent symptoms, decline of pulmonary function, and impaired quality of life; however, the disease can also follow a fulminant course with acute respiratory failure (*2*,*14*). Establishing a diagnosis of pulmonary disease due to *M. abscessus* complex is not straightforward because isolation of *M. abscessus* complex from respiratory samples is not, in and of itself, diagnostic of pulmonary disease (*2*). According to guidelines published by the American Thoracic Society/Infectious Diseases Society of America in 2007, the diagnosis of *M. abscessus* complex pulmonary disease requires the fulfillment of clinical and microbiological criteria, such as the presence of clinical symptoms; radiographic evidence of lesions compatible with NTM pulmonary disease; appropriate exclusion of other diseases; and, in most circumstances, positive culture results from at least 2 separate expectorated sputum samples (*2*). Common radiographic findings of *M. abscessus* complex pulmonary infection (i.e., bronchiolitis; bronchiectasis; nodules; consolidation; and, less frequently, cavities) are shown in Figure 3 (*2*).

*M. abscessus* complex is especially prevalent in respiratory specimens from patients with cystic fibrosis (*7*,*15*). Recent studies have shown that *M. abscessus* complex infection is no longer a contraindication for lung transplantation, although postoperative complications and a prolonged treatment course can be expected (*7*).

Pulmonary disease caused by *M. abscessus* complex is notoriously difficult to treat. Although there is no standard treatment, current guidelines suggest the administration of macrolide-based therapy in combination with intravenously administered antimicrobial agents; however, this regimen has been shown to have a substantial cytotoxic effect (*2*). Of 65 patients with pulmonary disease due to *M. abscessus* complex who received an initial 4-week course of intravenous antimicrobial agents followed by macrolide-based combination therapy, 38 (58%) had *M. abscessus*–negative sputum samples >12 months after treatment (*16*). Surgical resection of localized disease in addition to antimicrobial therapy has been shown to elicit a longer microbiologic response than antimicrobial agents alone: sputum samples were *M. abscessus* complex–negative for at least 1 year in 57% versus 28% of these treatment groups, respectively (*17*). According to the 2007 American Thoracic Society/Infectious Diseases Society of America guidelines, the treatment options remain limited with current antimicrobial agents, and *M. abscessus* complex pulmonary disease is still considered a chronic incurable disease (*2*).

The advancement of subspecies differentiation has allowed for more effective management of pulmonary disease caused by *M. abscessus* complex. For example, unlike *M. abscessus* subsp. *abscessus*, *M. abscessus* subsp. *massiliense* does not have inducible resistance to clarithromycin (*7*). Therefore, knowing that a patient’s infection is due to *M. abscessus* subsp. *massiliense* rather than 1 of the other 2 subspecies enables the physician to confidently administer clarithromycin (*7*,*18*). In a large study on treatment outcome in patients with pulmonary disease caused by *M. Abscessus* subsp. *massiliense* or *M. abscessus* subsp. *abscessus*, all patients had similar clinical signs, radiographic findings, and treatment regimens (*18*). However, after treatment, the percentage of patients who had negative sputum culture results was much higher in the *M. abscessus* subsp. *massiliense*–infected group (88%) than in the *M. abscessus* subsp. *abscessus*–infected group (25%) (*18*). The lack of efficacy of clarithromycin-containing antimicrobial therapy against *M. abscessus* subsp. *abscessus* isolates in the study could be explained by the subspecies’ inducible resistance to clarithromycin. The study clearly demonstrated how *M. abscessu*s subsp. *massiliense* and *M. abscessus* subsp. *abscessus* have different susceptibility profiles to combination therapy containing clarithromycin and different outcomes from such treatment (*18*).

### SSTIs

**Figure 4:**
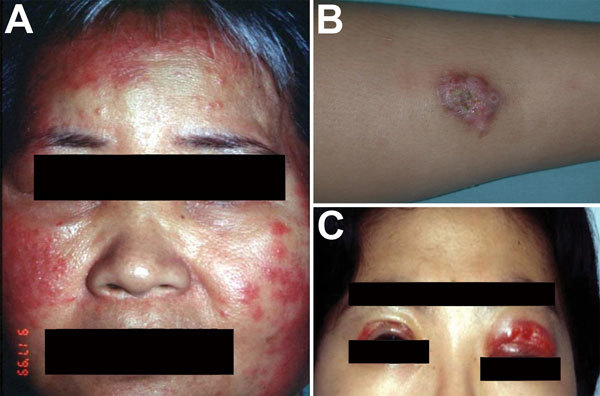
Skin lesions caused by *Mycobacterium abscessus* subsp. *abscessus*. A) Diffuse erythematous papular eruptions on the face and bilateral cervical lymphadenitis in a middle-aged man. B) A circumscribed subcutaneous nodule with pus discharge on the right arm of a 12-year-old boy. C) Wound infection over both upper eyelids of a 36-year-old woman; the infection developed 1 week after cosmetic surgery.

SSTIs are also commonly caused by *M. abscessus* complex; infections range from deep tissue infections to localized skin infections. The 2 major mechanisms for acquiring an *M. abscessus* complex–associated SSTI are by 1) direct contact with contaminated material or water through traumatic injury, surgical wound, or environmental exposure and 2) secondary involvement of skin and soft tissue during disseminated disease (*19*). SSTIs caused by *M. abscessus* complex have been reported in patients who recently underwent cosmetic procedures (e.g., mesotherapy), tattooing, and acupuncture (*19*). *M. abscessus* complex SSTIs can also develop after exposure to environmental sources, such as spas and hot springs (*19*,*20*). More often, however, these SSTIs develop among hospitalized postsurgical patients, in whom surgical wound infections are most commonly due to *M. abscessus* subsp. *massiliense* (*21*,*22*). Disseminated *M. abscessus* complex infections with skin and soft tissue involvement also commonly occur (*23*). Of note, however, the presence of *M. abscessus* complex SSTIs can result in or from disseminated *M. abscessus* complex infections (*23*). *M. abscessus* complex skin infection have diverse presentations, including cutaneous nodules (usually tender), erythematous papules/pustules, and papular eruptions or abscesses (Figure 4) (*19*).

### Central Nervous System Infections

**Figure 5:**
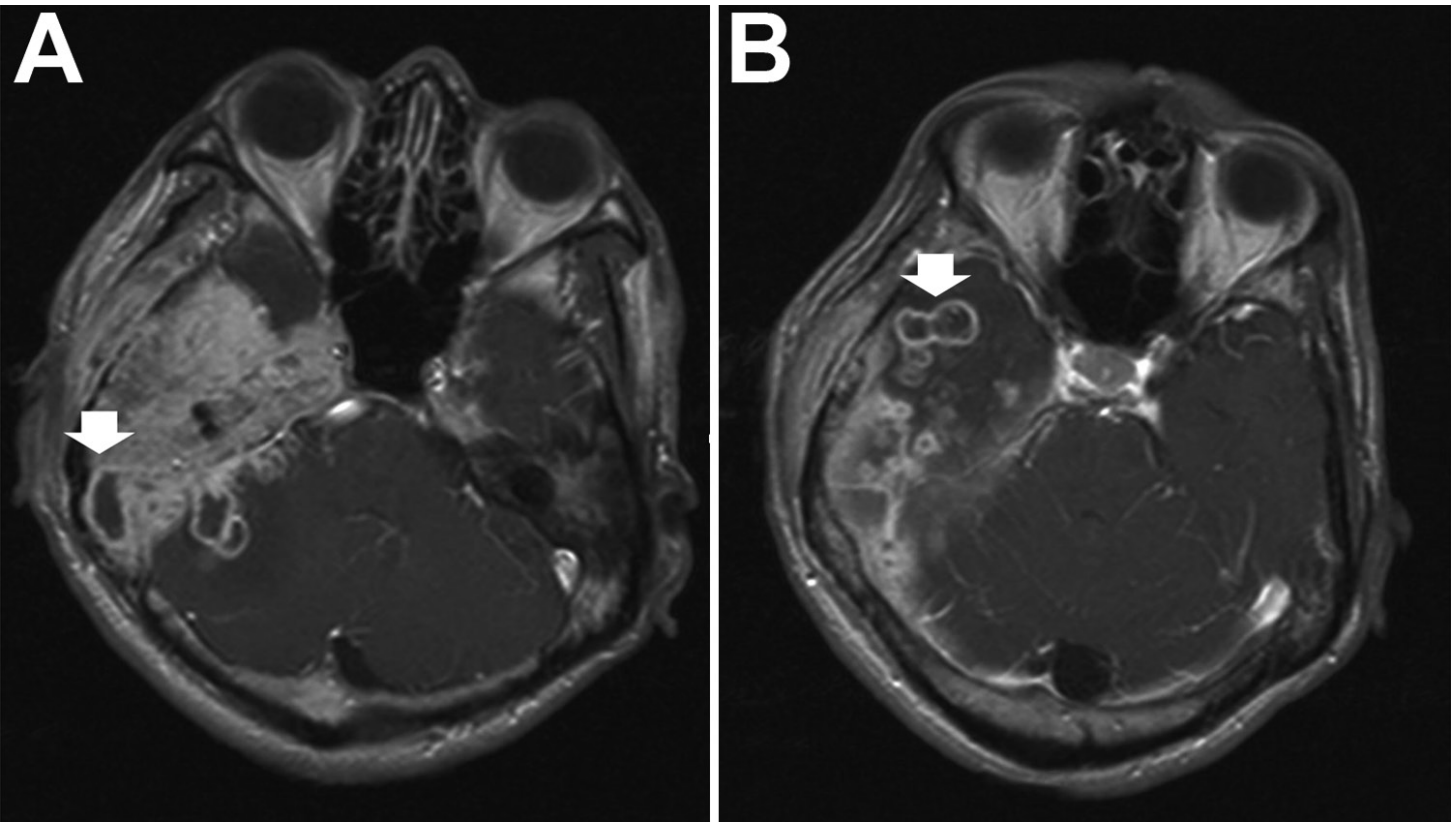
Brain computed tomography scan images for a patient with central nervous system infection caused by *Mycobacterium abscessus* subsp. *bolletii*. Arrows indicate abnormal nodular pachymeningeal thickening and leptomeningeal and intraparenchymal extension with multiple rim-enhancing lesions in the right cerebellum (A) and right temporal lobe (B), indicating cerebral abscesses.

Central nervous system (CNS) infections caused by *M. abscessus* complex are rare, but when they do occur, meningitis and cerebral abscesses are the most common manifestations (Figure 5). Although MAC is responsible for most NTM CNS infections, especially in HIV-infected hosts, *M. abscessus* complex has increasingly been reported to cause CNS infections in HIV-negative patients (*21*). In one study, *M. abscessus* was responsible for most NTM CNS infections in HIV-seronegative patients (8/11 patients), especially in patients who had undergone neurosurgical procedures, patients who had intracranial catheters, and patients with otologic diseases. Treatment outcome depended on the patient’s underlying disease and health status. Clarithromycin-based combination therapy for at least 1 year plus surgical intervention, if needed, offered the best chance for cure (*21*).

### Disseminated Diseases and Bacteremia

Disseminated *M. abscessus* complex infections, such as lymphadenopathy, SSTIs, pulmonary infections, and bacteremia, are on the rise (*23*), and bacteremia caused by *M. abscessus* complex is most often associated with catheter use (*24*,*25*). A recent study showed that surgical wound infection may be the portal of entry, especially for *M. abscessus* subsp. *massiliense* (*26*). Optimal treatment modalities include removal of intravascular catheters, surgical debridement, and administration of intravenous antimicrobial agents chosen on the basis of drug susceptibility test results.

Disseminated *M. abscessus* complex infections tend to occur in immunocompromised hosts, including persons with HIV. However, these infections can also occur in HIV-negative patients. Browne et al. (23) recently showed that neutralizing anti–interferon-γ autoantibodies were present in 81% of HIV-negative patients with disseminated NTM-associated infections, and in adults, these antibodies were associated with adult-onset immunodeficiency similar to that seen in advanced HIV infection. This adult-onset immunodeficiency status can lead to disseminated NTM disease that mimics advanced HIV infection (*23*).

### Ocular Infections

The incidence of NTM ocular infections (keratitis, endophthalmitis, scleritis, and other tissues of the ocular area) has increased over the past decade, and the increase has been attributed to the *M. chelonae/abscessus* group (*27*). Interpreting the real trend in ocular infections caused by *M. abscessus* complex is difficult because most studies have not used reliable tests to differentiate between *M. abscessus* complex and *M. chelonae* (*27*).

Initial treatment of *M. abscessus* complex ocular infections involves the discontinuation of topical corticosteroids, if used. The optimal treatment strategy (topical therapy, systemic antimicrobial agents, and surgical intervention) depends on the site of the ocular infection (*28*). Topical therapy, particularly topical amikacin and clarithromycin, can be used to treat some *M. abscessus* complex ocular infections (e.g., conjunctivitis, scleritis, keratitis, endophthalmitis) (*28*), and systemic antimicrobial agents can be used for all ocular infections (*28*). Surgical debridement, including removal of infected tissue, should be considered and is necessary for treatment of infections in some patients (*28*). Treatment outcome varies according to the site of infection, and early recognition of the infection is crucial.

## Nosocomial Outbreaks and Transmission

Outbreaks of *M. abscessus* complex infections in hospital and clinic settings have been reported worldwide (*19*). Many of the outbreak events occur in clinics conducting cosmetic surgery, liposuction, mesotherapy, or intravenous infusion of cell therapy (*29*). Proposed sources of transmission include contaminated disinfectants, saline, and surgical instruments as well as contact transmission between patients (*19*,*30*,*31*).

*M. abscessus* complex transmission involves vulnerable hosts and causes substantial illness and death; thus, concern is also rising regarding outbreaks in centers specializing in lung transplantation and treatment of cystic fibrosis (*30*). Whole-genome sequencing of outbreak isolates has provided evidence of patient-to-patient transmission of *M. abscessus* complex; this transmission is most likely indirect rather than direct (*30*).

## Antimycobacterial Susceptibilities

*M. abscessus* complex is notoriously resistant to standard antituberculous agents and most antimicrobial agents (*5*). The Clinical and Laboratory Standards Institute recommends testing rapidly growing mycobacteria for susceptibility to macrolides (clarithromycin and amikacin), aminoglycosides, fluoroquinolones, imipenem, doxycycline, tigecycline, cefoxitin, cotrimoxazole, and linezolid (*32*). The recommended drug susceptibility testing method is broth microdilution in cation-adjusted Mueller-Hinton broth supplemented with oleic albumin dextrose catalase (*32*). Among the agents suggested for *M. abscessus* complex susceptibility testing, clarithromycin, amikacin, and cefoxitin have the best in vitro antimycobacterial activity (*7*,*32*,*33*).

Recent major studies presenting susceptibility and resistance rates for *M. abscessus* subsp. *massiliense*, *M. abscessus* subsp. *abscessus*, and *M. abscessus* complex against 7 antimicrobial agents are summarized in Table 1. Most of the studies are from Asia, and the resistance rate for clarithromycin ranges from 0 to 38%. The resistance rates for cefoxitin (overall 15.1%) and amikacin (overall 7.7%) are also low. Doxycycline, quinolones (including moxifloxacin and ciprofloxacin), and imipenem had high resistance rates. Therefore, local susceptibility data are needed to guide treatment.

**Table 1:** Summary of recent data on the resistance of *Mycobacterium abscessus* complex bacteria to different antimicrobial agents*

Study authors (reference), species	No. isolates	Antimicrobial drug, no. resistant isolates/no. tested (%)						
		CLR	DOX	CIP	MXF	FOX	AMK	IPM
Lee et al. (*34*)								
*M. abscessus* subsp. *abscessus*	202	48/202 (24)	NA	184/202 (91)	167/202 (83)	NA	25/202 (12)	NA
*M. abscessus* subsp. *massiliense*	199	15/199 (8)	NA	174/199 (87)	149/199 (75)	NA	12/199 (6)	NA
Koh et al. (*18*)								
*M. abscessus* subsp. *abscessus*	64	3/64 (5)	53/64 (83)	37/64 (58)	30/64 (47)	0/64	3/64 (5)	27/62 (44)
*M. abscessus* subsp. *massiliense*	79	3/79 (4)	58/79 (73)	48/79 (61)	42/79 (53)	1/79 (1)	6/79 (8)	50/75 (67)
Huang et al. (*35*)								
*M. abscessus* complex	40	3/40 (8)	37/40 (93)	36/40 (90)	31/40 (78)	27/40 (68)	2/40 (5)	35/40 (88)
Brown-Elliott et al. (*36*)								
*M. abscessus* complex	37	0% (0/37)	NA	29/37 (78)	29/37 (78)	NA	0/37	7/37 (19)
Broda et al. (*37*)								
*M. abscessus* complex	58	22/58 (38)	57/58 (98)	55/58 (95)	55/58 (95)	16/58 (28)	10/58 (17)	56/58 (97)
Zhuo et al. (*38*)								
*M. abscessus* complex	70	10/70 (14)	NA	56/70 (80)	NA	3/70 (4)	0/70	15/70 (21)
Overall								
*M. abscessus* complex	749	104/749 (13.9)	205/241 (85.1)	619/749 (82.6)	503/679 (74.1)	47/311 (15.1)	58/749 (7.7)	190/342 (55.6)
*M. abscessus* subsp. *abscessus*	266	51/266 (19.4)	53/64 (83.0)	221/266 (83.1)	197/266 (74.1)	0/64	28/266 (10.5)	27/62 (44.0)
*M. abscessus* subsp. *massiliense*	278	18/278 (6.5)	58/79 (73.4)	222/278 (79.8)	191/278 (68.7)	1/79 (1.0)	18/278 (6.5)	50/75 (66.7)

*AMK, amikacin; CIP, ciprofloxacin; CLR, clarithromycin; DOX, doxycycline; FOX, cefoxitin; IPM, imipenem; MXF, moxifloxacin; NA, not available.

Because of its rarity, *M. abscessus* subsp. *bolletii* is discussed separately here. These mycobacteria are uniformly resistant to drugs recommended for use against *M. abscessus* complex. In one study, high MICs of tested antimycobacterial agents were observed, and amikacin probably had the highest activity (i.e., the lowest MIC) (*33*).

Recent studies have reported on the importance of the *erm*(41) gene in *M. abscessus* complex; this gene confers macrolide resistance through methylation of 23S ribosomal RNA (*39*). The *erm*(41) gene is present in the *M. abscessus* complex group but absent in *M. chelonae* (*39*). Many strains of *M. abscessus* subsp. *massiliense* have a nonfunctional *erm*(41) gene, and because of this, the rate of clarithromycin susceptibility is higher in *M. abscessus* subsp. *massiliense* than in *M. abscessus* subsp. *abscessus* (*18*). The Clinical and Laboratory Standards Institute recommends testing for inducible macrolide resistance because subspecies of *M. abscessus* complex demonstrate susceptibility to clarithromycin during the first 3–5 days of incubation but demonstrate resistance after an extended duration of incubation (preferably 14 days, according to many experts) (*39*).

Another area of strenuous clinical research involves identifying and developing novel anti–*M. abscessus* complex agents. One such agent, the glycylcycline tigecycline, has been shown to exhibit good in vitro activity against rapidly growing mycobacteria, especially *M. abscessus* complex (*12*,*21*). However, no prospective trial has been conducted to evaluate the efficacy of tigecycline, and a breakpoint for interpreting tigecycline susceptibility has not been established (*32*).

## Treatment

Several problems regarding treatment of *M. abscessus* complex infections in different organs are unsolved. For example, there is a lack of consensus on the optimal antimicrobial agents and combination therapy, optimal treatment duration, and the introduction of novel antimicrobial agents (e.g., tigecycline). Reports describing cases of *M. abscessus* complex infection are limited, except for those describing pulmonary disease and SSTIs. Thus, treatment recommendations must rely on retrospective case series. A summary of treatment recommendations from previous studies is shown in Table 2. The treatment of serious *M. abscessus* complex disease usually involves initial combination antimicrobial therapy with a macrolide (clarithromycin 1,000 mg daily or 500 mg twice daily, or azithromycin 250 mg–500 mg daily) plus intravenous agents for at least 2 weeks to several months followed by oral macrolide–based therapy (*2*). The drugs of choice for initial intravenous administration are amikacin (25 mg/kg 3×/wk) plus cefoxitin (up to 12 g/d given in divided doses) or amikacin (25 mg/kg 3×/wk) plus imipenem (500 mg 2–4×/wk) (*2*). As previously mentioned, the in vitro MICs of tigecycline are low, and the drug should be considered in treatment regimens.

**Table 2:** Summary of recommendations from previous studies for the treatment of *Mycobacterium abscessus* complex infections in humans

Type of disease (reference)	Recommended initial regimen	Recommended treatment duration
Pulmonary disease (*2*)	Macrolide-based therapy in combination with intravenous antimicrobial therapy (preferably cefoxitin and amikacin)	Continue until sputum samples are negative for *M. abscessus* complex for 12 mo
Skin and soft-tissue infection (*2*)	Macrolide in combination with amikacin plus cefoxitin/imipenem plus surgical debridement	Minimum of 4 mo, including a minimum of 2 wk combined with intravenous agents
Central nervous system infection (*21*)	Clarithromycin-based combination therapy (preferably including at least amikacin in the first weeks)	12 mo
Bacteremia (*24**,**25*)	At least 2 active antimicrobial agents (preferably including amikacin) plus removal of catheter and/or surgical debridement of infection foci	4 wk after last positive blood culture result
Ocular infection (*28*)	Topical agents (amikacin, clarithromycin) and/or systemic antimicrobial drugs (oral clarithromycin, intravenous amikacin or cefoxitin) and/or surgical debridement*	6 wk to 6 mo

*The treatment of ocular infections was highly dependent on the infection site. In some sites, *>*1 treatment strategies (i.e., topical or systemic antimicrobial drug treatment or surgery) should be considered.

## Prevention

*M. abscessus* complex infection can be acquired in the community or in the hospital setting. In the community setting, water supply systems have been postulated to be the source of human infections (*7*,*40*). Membrane filtration, hyperchlorination, maintenance of constant pressure gradients, and the utilization of particular pipe materials have been suggested as methods for reducing the presence of NTM in water supply systems (*7*,*40*). In the hospital setting, disinfectant failure, contamination of medical devices and water, and indirect transmission between patients are considered to be the source of infections (*19*,*30*). In addition, clinics for cosmetic procedures have become sites of frequent outbreaks of *M. abscessus* complex infections (*19*,*29*). It is unclear whether patients with *M. abscessus* complex disease should be isolated from vulnerable hosts, such as patients with cystic fibrosis.

## Conclusions

*M. abscessus* complex comprises a group of rapidly growing, multidrug-resistant, nontuberculous mycobacteria that are responsible for a wide spectrum of SSTIs and other infections. The complex is differentiated into 3 subspecies: *M. abscessus* subsp. *abscessus, M. abscessus* subsp. *massiliense*, and *M. abscessus* subsp. *bolletii*, which is rarely isolated. The major difference between *M. abscessus* subsp. *massiliense* and *M. abscessus* subsp. *abscessus* is that the former does not have an intact *erm*(41) gene and thus does not have inducible macrolide resistance; treatment response may thus be better among patients with infections caused by *M. abscessus* subsp. *massiliense*. *M. abscessus* complex can cause infections involving almost all organs, but the infections generally involve the lungs, skin, and soft tissue. Drugs with the best in vitro activity include clarithromycin, amikacin, cefoxitin, and possibly tigecycline. Treatment regimens vary according to the infection site and usually include macrolide-based combination therapy, including parenteral amikacin plus another parenteral agent (cefoxitin, tigecycline, imipenem, or linezolid), for weeks to months, followed by oral antimicrobial therapy. Evidence of nosocomial transmission and outbreaks of *M. abscessus* complex is increasing; therefore, strenuous infection control measures should be taken to reduce the possibility of hospital-acquired *M. abscessus* complex infections.

Because of the complexity of the molecular techniques needed to differentiate between *M. abscessus* subsp. *abscessus* and *M. abscessus* subsp.* massiliense*, it is difficult for most laboratories to identify the different subspecies. A more rapid and less expensive method for subspecies identification is thus needed for epidemiologic and clinical purposes. In addition, prospective trials comparing different regimens of antimicrobial agents are needed to determine the best treatment options; these studies should include novel agents, such as tigecycline. The effect of implementing isolation protocols for patients with infections due to *M. abscessus* complex (particularly pulmonary disease) should also be evaluated in future studies.
